# Study on quantitative expression of PPARγ and ADRP in muscle and its association with intramuscular fat deposition of pig

**DOI:** 10.1186/s40064-016-3187-0

**Published:** 2016-09-07

**Authors:** Jingxiang Cui, Wei Chen, Jie Liu, Tao Xu, Yongqing Zeng

**Affiliations:** 1 College of Animal Science and Technology, Shandong Agricultural University, Taian, 271018 China; 2 Weifang University of Science and Technology, Shouguang, 262700 Shandong China; 3 Key Laboratory of Plant Resources, Institute of Botany, Chinese Academy of Sciences, Beijing, 100093 China

**Keywords:** Pig, PPARγ, ADRP, QRT-PCR, Fat deposition

## Abstract

**Background:**

Intramuscular fat (intramuscular fat, IMF) is one of the important traits of pork quality. How to reasonably improve the intramuscular fat content is the most focus researchers. Some possible regulation of intramuscular fat deposition of candidate genes to cause the attention of people. The objective of this study was to elucidate the relationship between peroxisome proliferator-activated receptor γ (PPARγ) and adipose differentiation-related protein (ADRP) mRNA expression and intramuscular fat (IMF) deposition in the muscle tissue of three breeds of pig: Laiwu (LW), Lulai Black (LL), and Large White (LY).

**Results:**

qPCR analysis of the PPARγ and ADRP genes in the three breeds of pig revealed PPARγ and ADRP mRNA expression profiles of LW > LL > LY and LL > LW > LY, respectively. PPARγ mRNA expression was significantly and positively correlated with IMF deposition (*p* < 0.05). There were significant correlations between PPARγ and ADRP mRNA expression levels (*p* < 0.01).

**Conclusions:**

These results suggest correlations between PPARγ and ADRP in fat deposition and regulation in pigs, PPARγ gene may be a main effector of IMF content and play an important role during adipocyte differentiation in pigs, thereby providing new information to further elucidate molecular mechanisms associated with intramuscular fat deposition in Laiwu pigs and provides new data for further molecular studies of mechanisms underlying intramuscular fat deposition in human obesity. The continued elucidation of specific genetic mechanisms between PPARγ and ADRP warrants further studies.

## Background

Intramuscular fat (IMF) content is an important trait to determine pig meat quality and has been positively correlated with meat tenderness, moisture content, and taste (Wood et al. 2004; Lu et al. 2008; Hausman et al. 2009). Although several candidate genes play only minor roles, together they construct the genetic basis determining fat deposition. Therefore, the search for candidate genes and molecular mechanisms associated with IMF deposition has become an important strategy in IMF research. At present, much research has focused on the identification of candidate genes and underlying mechanisms associated with the regulation of porcine intramuscular fat deposition, with the goal to reasonably improve IMF content.

Peroxisome proliferator-activated receptor γ (PPARγ), as a candidate gene, is a ligand activated transcription factor and a member of the nuclear hormone receptor superfamily (Bispham et al. 2005). Previous research has shown that PPARγ, which is predominantly expressed in adipose tissue and the liver, is a master regulator of adipogenesis and controls adipocyte differentiation, proliferation, and lipid accumulation (He et al. 2013). In addition, PPARγ regulates the expression of genes associated with sugar metabolism and lipid biosynthesis. Recent researches indicate that progranulin (PGRN) is closely related to diabetes and is regarded as a novel adipokine associated with obesity development, affecting adipocyte biology. Also, PPARγ activity can be adjusted by various methods and materials to increase PGRN polypeptide levels (Yang et al. 2013).

Adipose differentiation-related protein (ADRP) is covered in a phospholipid protein lipid droplet surface and can up-regulate fatty acid production (Imamura et al. 2002; Robenek et al. 2006) and promote the formation of lipid droplets for storage of fatty acids (Listenberger et al. 2007).

Previous studies have shown that the PPARγ and ADRP genes play important roles in the regulation of animal lipid deposition. PPARγ activity can be adjusted by various methods and materials, ADRP can up-regulate fatty acid production. Therefore, the objective of the present study was to elucidate the relationship between PPARγ and ADRP mRNA expression and IMF deposition in muscle and to identify the major genes that regulate IMF deposition to improve IMF content in pork.

Laiwu Pig, an excellent type of North China local fatty breed of pig, is known for high meat quality with good tenderness (Zeng et al. 2005), bright color, and higher IMF, while it is the best animal model to study the mechanisms of high intramuscular fat deposition (Spurlock and Gabler 2008; Chen et al. 2013). The Lulai Black pig is a cross of a Laiwu pig with introgression of a Large White pig. As the low intramuscular fat pig animal model, Large Whites (LY) is the best characterized and studied breed of pig (Grzes et al. 2016; Davoli et al. 2015; De Rosa et al. 2013).

This study is the first to investigate PPARγ and ADRP gene expression profiles in Laiwu pigs and to report the effects of PPARγ and ADRP mRNA expression on regulation of fat deposition in three breeds of pig.

## Methods

### Experimental animals

This work was approved by the Institutional Animal Care and Use Ethics Committee of Shandong Agricultural University and carried out in accordance with the “Guidelines for Experimental Animals” of the Ministry of Science and Technology (Beijing, PR China). The experimental cohort consisted of 30 castrated boars composed of equal numbers of three breeds: Laiwu (LW), Lulai Black (LL), and Large Whites (LY). All pigs were housed at Laiwu Breeder Pig Farm Co. Ltd. (Laiwu city, Shandong Province, China) and fed a diet formulated to meet current nutritional requirements (NRC 1998). On the day of slaughter, the mean weight of the pigs was 114 ± 2 kg. Samples were collected from the *longissimus dorsi* muscle at the last rib and a portion of the muscle tissue. Afterward, the remaining samples were stored at −80 °C for further analysis.

### IMF content measurement

IMF was chemically quantified following the ISO, 1443-1973. The method used was direct Soxhlet extraction of fat by a solvent (AOAC 2000).

### RNA extraction and cDNA preparation

Total RNA was extracted from the muscle samples using TRIZOL^®^ Reagent (Invitrogen, San Diego, CA, USA) and its integrity was verified in a 1 % agarose gel. Total RNA was reverse transcribed to cDNA using SuperScrit TM III Reverse Transcriptase (Invitrogen) according to the manufacturer’s instructions (Omi et al. 2005; Cui et al. 2011).

### Primer design

Primers for amplification of PPARγ (GenBank_NM214379.1) and ADRP (GenBank_NM214200.1) mRNA were designed using Premier 5.0 software (PREMIER Biosoft, Palo Alto, CA, USA). The primer pair used for amplification of ACTB mRNA is reported elsewhere (AOAC 2000). All primer sequences are listed and described in detail in Table 1, and were synthesized by Sangon Biotech (Shanghai) Co., Ltd. (Shanghai, China).

**Table 1:** Primer sequences used in this study

Genes	Primer sequence (5′–3′)	Product length (bp)	Anneal temp (°C)
*PPARγ*	Forward: TCCAGCATTTCCACTCCACACT	207	58
	Reverse: GAATAAGGCGGGGACACAG		
*ADRP*	Forward: TTTGCCAGGAAGAATGTGC	255	58
	Reverse: TGGTAACCCTCGGATGTTG		
*ACTB*	Forward: TCTGGCACCACACCTTCT	120	58
	Reverse: TGATCTGGGTCATCTTCTCAC		

### Establishment of a real-time fluorescence quantitative PCR (qPCR) system

SYBR Green real-time PCR amplification was conducted using an Mx3000PTM System (Stratagene Corporation, Santa Clara, CA, USA).The optimal reaction system and conditions were determined by experimentation. The continuously expressed gene, ACTB, was used as an endogenous reference for determination of targeted mRNA profiles. Each qPCR amplification was performed in a 15-μL reaction volume consisting of 7.5 μL of SYBR^®^ Permix Ex Taq™ (TaKaRa, Japan) spiked with 0.3 μL of ROX Reference Dye II (TaKaRa), cDNA 5 μL of PPARγ and ADRP, forward and reverse primers, and 0.3 μL of PPARγ (10 μmol/L) and ADRP (10 μmol/L), respectively, and supplemented with water to a final volume of 15 μL. The following amplification conditions were used: one cycle of 15 s at 95 °C, followed by 40 cycles of 10 s at 95 °C, 20 s at 58 °C, and a final extension for 15 s at 72 °C. The ACTB gene was used as an internal control in the reaction system and amplified in triplicate in accordance with the conditions described above.

### Statistical analysis

The results were analyzed using Mx3000P quantitative analysis software and relative quantitative results were calculated using the adjusted $2^{{ - {\Delta \Delta }{\text{C}}_{\text{T}} }}$ method (Erkens et al. 2006; Livak and Schmittgen 2001). Differential gene expression in muscle tissues collected from the three breeds was subjected to bivariate correlation analysis with repeated measures. Correlation analysis between PPARγ and ADRP mRNA expression levels and meat quality were calculated using the PROC CORR procedure included with the SAS ver. 8.1 software package (SAS Institute, Cary, NC, USA). The results are presented as means ± the standard error of the mean. Probability (*p*) values of <0.05 and <0.01 were considered significant and extreme differences, respectively.

## Results

### IMF content measurement

IMF content was chemically quantified in a total of 30 pigs in accordance with the ISO 1443-1973 protocol with direct Soxhlet extraction of fat by a solvent. As shown in Table 2, the average IMF content of LW was significantly higher than that of LL and LY, indicating significant differences among breeds (*p* < 0.05).

**Table 2:** The average intramuscular fat content in three breeds of pig

	LW	LL	LY
IMF (%)	15.94 ± 1.8^a^	3.63 ± 0.82^b^	1.15 ± 0.1^c^

The data are expressed as mean ± standard error

Letters denote the difference of IMF content with significantly different (*p* < 0.05)

*LW* Laiwu Black, *LL* Lulai Black, *LY* Large White pig

### PPARγ and ADRP mRNA expression level

**Fig. 1:**
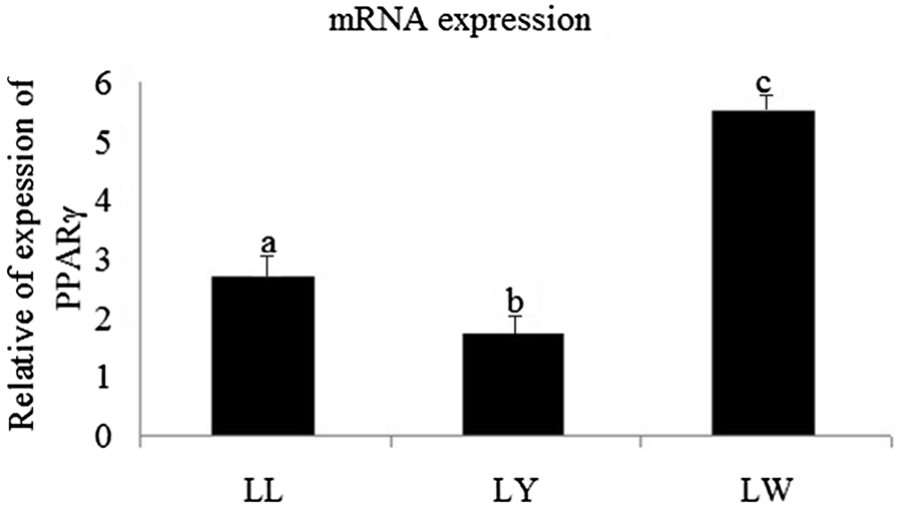
Relative abundance of PPARG mRNAs in muscle tissues of three breeds pig

**Fig. 2:**
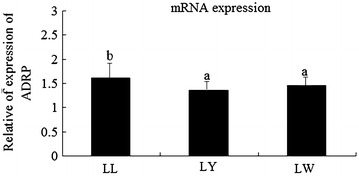
Relative abundance of ADRP in muscle tissues of three breeds pig

As shown in Fig. 1, there were significant differences in PPARγ mRNA expression levels among breeds (*p* < 0.05), with highest values in LW followed by LL and LY. Also, there was significant difference in ADRP mRNA expression levels among breeds, with highest values in LL, followed by LY and LW (*p* > 0.05) (Fig. 2).

### PPARγ and ADRP mRNA expression correlated with IMF content

The results of correlation analysis between PPARγ and ADRP mRNA expression with IMF content are shown in Table 3. PPARγ mRNA expression in muscle was positively correlated with IMF (*p* < 0.05) in all three breeds of pig. ADRP mRNA expression in muscle was not correlated with IMF. With regard to IMF, LW was significantly thicker than LY, there were significant differences between LW and LL. These results imply that the ADRP gene is not influenced by a variety of factors, while PPARγ expression was significantly correlated with IMF content (*p* < 0.01).

**Table 3:** Correlation coefficients between PPARγ, ADRP expression and IMF content

	PPARγ	ADRP
IMF	0.58*	0.09
PPARγ	–	0.74**
ADRP	0.74**	–

* *p* < 0.05; ** *p* < 0.01

## Discussion

### PPARγ and ADRP mRNA expression

The results of this study showed that mRNA levels of PPARγ and ADRP in the muscle tissues of the three breeds of examined pigs occurred in the following orders, LW > LL > LY and LL > LW > LY, respectively. PPARγ belongs to a nuclear receptor superfamily of transcription factors and plays a critical role in adipocyte differentiation and fat deposition in mammals (Farmer 2006; Zeng et al. 2012).

PPARγ mRNA expression was significantly correlated with IMF in muscle in each of the three breeds of pig, suggesting some type of relationship between the gamma component, which may be related to the fat deposition traits associated with PPAR. PPARγ is mainly involved in the induction of differentiation of adipose cells as well as activation of the expression of phosphoenolpyruvate and other fatty tissue-specific genes (Pan et al. 2014). Meanwhile, Ma et al. (2015) found that the PPARγ-BsrI loci can be used as a candidate gene loci of pork quality traits.

### PPARγ mRNA expression is correlated with IMF content

PPARγ mRNA expression in muscle was significantly and positively correlated with IMF (*p* < 0.05) in the three breeds of pig, further confirming that PPARγ plays a certain role in fat traits. Farmer (2006) and Kim et al. (2005) pointed out that the gamma component is extremely important in anomalies in the process of differentiation of preadipocytes and adipocyte maturation associated with the complex PPAR transcription factor, and can directly or indirectly regulate other transcription factors to induce activation of fat cells. The results of a study of Rongchang piglets showed that increased gene expression of PPARγ in adipose tissues may regulate fat deposition throughout the whole body (Bildirici et al. 2003). Another study reported that miRNA-130b suppressed fat deposition by inhibiting PPARγ expression and demonstrated a significant ability to down-regulate PPARγ expression, which was associated with reduced adipogenesis and lipogenesis in primary cultured porcine adipocytes (Rosen and Spiegelman 2001; Taniguchi et al. 2014).

Together, these results suggest that the PPARγ gene may be a main effector of IMF content and play an important role during adipocyte differentiation in pigs, thereby providing new information to further elucidate molecular mechanisms associated with intramuscular fat deposition in Laiwu pigs.

By confirming a functional pig muscle-specific promoter, we could drive fat-related gene overexpression in skeletal muscle and improve IMF content via the development of transgenic pigs.

### ADRP mRNA expression is not correlated with IMF content

There was no significant correlation between ADRP mRNA expression in muscle and IMF (*p* > 0.05) and there were significant correlations with PPARγ and ADRP mRNA expression levels (*p* < 0.01). ADRP plays an important role in regulating lipid storage in various cells. Kim et al. (2005) investigated ADRP as a candidate gene of intramuscular fat deposition and marbling traits in pigs. Results showed that, in the aspect of human studies (Yuan et al. 2008), ADRP gene expression was mediated by PPARγ. Also, PPARγ is a very important mediating factor in the resistance to insulin. Research of human trophoblasts showed that ADRP expression was regulated by PPARγ/Retinoid X receptor.

## Conclusions

These results suggest correlations between PPARγ and ADRP in fat deposition and regulation in pigs. PPARγ gene may be a main effector of IMF content and play an important role during adipocyte differentiation in pigs, thereby providing new information to further elucidate molecular mechanisms associated with intramuscular fat deposition in Laiwu pigs and provides new data for further molecular studies of mechanisms underlying intramuscular fat deposition in human obesity. The continued elucidation of specific genetic mechanisms between PPARγ and ADRP warrants further studies.

## Abbreviations

PPARγ: peroxisome proliferator-activated receptor γ
ADRP: adipose differentiation-related protein
IMF: intramuscular fat
LW: Laiwu pig
LL: Lulai Black pig
LY: Large White
PGRN: progranulin
